# Research on the Anisotropic Fracture Mechanical Properties of Shale Based on Three-Point Bending Experiments of Semi-Circular Disks

**DOI:** 10.3390/ma18071570

**Published:** 2025-03-30

**Authors:** Xinyue Wang, Lianke Cui, Lianzhi Yang, Fanmin He

**Affiliations:** 1 School of Civil and Resource Engineering, University of Science and Technology Beijing, Beijing 100083, China; 2 Petroleum Engineering Technology Research Institute, Sinopec Henan Oilfield Company, Nanyang 473132, China; 3 Chengdu Surveying Geotechnical Research Institute Co., Ltd. of MCC, Chengdu 610044, China

**Keywords:** semi-circular disk, central straight crack, bedding, anisotropy, fracture toughness, energy release rate

## Abstract

The three-point bending test is a key method for determining parameters related to the mechanical fracture properties of rocks. In this study, shale outcrops from Changning County, Sichuan Province, China, were selected. Three-point bending experiments were performed on shale semi-circular disks with a central straight crack, tested both perpendicular and parallel to the bedding direction. The corresponding load–displacement curves and crack opening displacements were obtained. The opening displacements of the specimens were measured through digital image technology, and the tensile strength and stiffness of the specimens were further calculated. A finite element model of the three-point bending test was developed. By integrating the finite element model with the experimentally obtained load–displacement curves, the anisotropic elastic moduli of the shale were inversely determined. Fracture toughness was calculated using two approaches: a formula from the International Society for Rock Mechanics and numerical methods using the finite element model, which was appropriately configured with the previously obtained elastic modulus values. The stress intensity factors for each specimen were calculated and compared. The energy release rate of shale was computed based on the fracture toughness. Results showed that both the fracture toughness and energy release rate of shale were greater in the perpendicular bedding direction than in the parallel direction. As an example, one specimen’s elastic modulus, opening displacement, and energy release rate obtained from experiments were input into the numerical simulation of the three-point bending test. The simulated load–displacement curve matched the experimental results well. This study provides a comprehensive approach to evaluating the anisotropic mechanical fracture properties of shale formations, which is essential for improving the accuracy of hydraulic fracture prediction models and enhancing the efficiency of shale gas extraction.

## 1. Introduction

Fracturing technology is widely applied in the oil and gas industry, particularly in shale oil and gas fields [1,2,3]. From a geomechanical perspective, the hydraulic fracturing process consists of three distinct phases: the initiation of fractures, the propagation of these fractures, and the subsequent flow-back stage [4]. The final fracture network is closely related to local geological conditions, such as rock properties and the distribution of natural fractures. Therefore, obtaining the anisotropic parameters of rocks is particularly important. Fracture toughness and energy release rate are key indicators for assessing the difficulty of crack propagation [5] and are critical factors in determining whether cracks can initiate successfully during the fracturing process [6].

The three-point bending test is an important method for determining the fracture toughness of rocks. The International Society for Rock Mechanics (ISRM) [7] has proposed several specimen types, including the short-rod (SR) specimen with V-shaped notches [8], the three-point bending circular-rod (CB) specimen with V-shaped notches [9], the cylindrical core with a central-notched Brazilian disk (CCNBD) specimen [10], the notched semi-circular bending (NSCB) specimen [11], and the cuboid specimen [12]. Among these, the NSCB three-point bending experiment is widely used due to its simple specimen preparation, ease of experimentation, and high measurement reliability (Aliha et al. [13], Zhang et al. [14], Wei et al. [15], Dia et al. [16]). Zhao et al. [17] compared the load–displacement curves and fracture toughness obtained from CCNBD and NSCB tests on marble and found that the NSCB results were more accurate. The NSCB specimens, with straight or V-shaped notches, are commonly used in dynamic fracture testing and in determining dynamic fracture toughness. Zhao et al. [18] studied the influence of notch width on fracture toughness using NSCB tests on marble. Wen et al. [19] analyzed the fracture toughness of NSCB specimens from Longmaxi Formation shale under various crack inclination angles.

The bedding structure of shale results in anisotropic mechanical fracture properties. Understanding the anisotropic fracture behavior of shale is essential for optimizing shale gas extraction strategies and improving efficiency [20,21,22,23,24,25]. Shi et al. [26] focused on the anisotropy of shale fracture toughness under different bedding angles using NSCB tests, finding that fracture toughness increases linearly with the bedding angle. Li et al. [27] found that the crack propagation direction is closely related to the angle between the notch and the bedding through three-point bending tests with varying notch-bedding angles. Chandler et al. [28] analyzed crack deflection conditions in shale and estimated its anisotropic fracture toughness. Zuo et al. [29] conducted three-point bending tests at different bedding dip angles to obtain fracture toughness. Liu [30] studied crack initiation, propagation, and failure modes in shale under three-point bending loads at different bedding angles using both experiments and phase-field simulations.

In this paper, straight NSCB tests were performed both perpendicular and parallel to the bedding to obtain load–displacement curves and crack opening displacements (CODs). The NSCB tests were designed to capture the anisotropic behavior of the specimens by conducting tests in both perpendicular and parallel orientations to the bedding planes. We obtained the elastic modulus of each specimen through inversion calculations. Using these curves, the fracture toughness and stress intensity factors of each specimen were calculated, and the anisotropic energy release rate was derived using a finite element model of NSCB. The results were applied to model the three-point bending process. Numerical simulation results were compared with experimental data to verify the accuracy of the fracture toughness and energy release rate calculations.

## 2. Shale Three-Point Bending Experiments

### 2.1. Specimen Preparation

**Figure 1:**
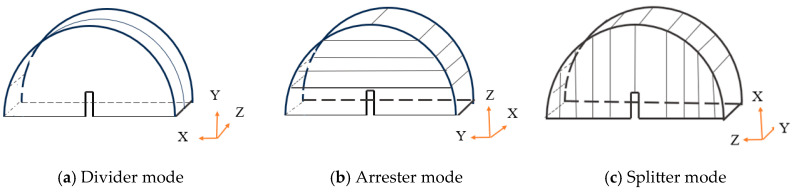
Shale coring methods and NSCB specimens.

The shale outcrop in Changning City, China, was chosen for this study. Given the distinct bedding characteristics of shale, cores were drilled both parallel and perpendicular to the bedding. Three groups of shale NSCB specimens were prepared, as illustrated in Figure 1a–c, based on the orientation of the notch relative to the bedding plane. In Figure 1, the coring direction of the disk perpendicular to the bedding (Figure 1a) is referred to as the “divider”. The disks for the “arrester” (Figure 1b) and “splitter” (Figure 1c) modes were cored parallel to the bedding, with angles between the notch direction and the bedding plane of 0° and 90°, respectively.

Following the specimen preparation guidelines outlined by ISRM [31], 8 specimens were prepared for each mode using the cutting methods shown in Figure 1. The specimens were named according to the following scheme: the first letter “N” represents the NSCB specimen type; the second letter “d”, “a”, or “s” denotes the three modes (divider, arrester, and splitter); and the third letter “h” or “v” corresponds to the horizontal or vertical coring direction. Thus, the 24 specimens were named as follows: the divider specimens in Figure 1a are Ndv 1–8, the arrester specimens in Figure 1b are Nah 1–8, and the splitter specimens in Figure 1c are Nsh 1–8. The physical parameters of all specimens are listed in Table 1. The shale specimens were stored and tested under strict environmental controls with the temperature maintained around 20 °C and the humidity kept at 20% RH.

### 2.2. Specimen Testing Methods

**Figure 2:**
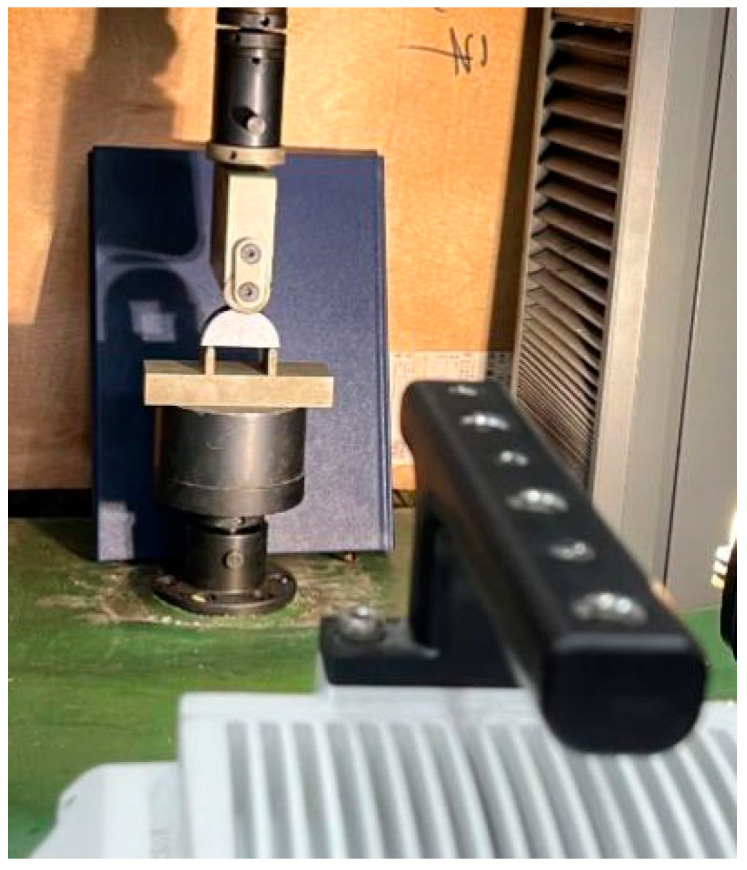
Diagram of the loading configuration and testing equipment for NSCB specimens.

For the prepared NSCB specimens, a support frame was selected based on ISRM recommendations [32]. The distance between the support points was set to 40 mm, and the radius of the supporting parts was 5 mm. A TFD-20D testing machine (Changchun, China) was used for the three-point bending experiments, with a maximum load capacity of 100 KN and a relative error of 1%. The specimens were loaded at a constant displacement rate of 0.1 mm/min, which meets the requirement for static crack propagation (loading rate should not exceed 0.2 mm/min) [33]. A Phantom UHS-12V2012 high-speed camera (Shanghai, China) recorded the experimental process. The overall loading configuration for the three-point bending experiment of NSCB specimens is shown in Figure 2.

**Figure 3:**
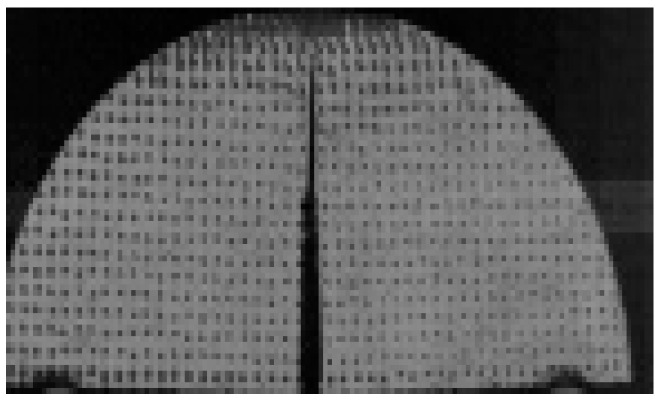
A NSCB specimen smeared by scatter points.

Digital image measurement is a proven, cost-effective, and accurate method for studying deformation in cracked specimens [34]. Scatter points were applied to all NSCB specimens, as shown in Figure 3. Displacement fields during loading were obtained using Digital Image Correlation (DIC) technology.

### 2.3. Load–Displacement Curves

**Figure 4:**
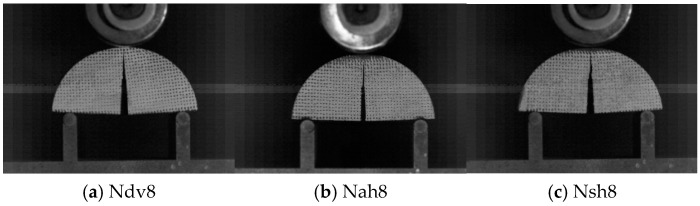
Crack propagation images.

Figure 4a–c show images of crack propagation in the Ndv, Nah, and Nsh specimens, captured by the high-speed camera. It is evident from these images that, regardless of bedding orientation, cracks initiate along the loading direction and propagate vertically along the direction of the pre-existing cracks.

**Figure 5:**
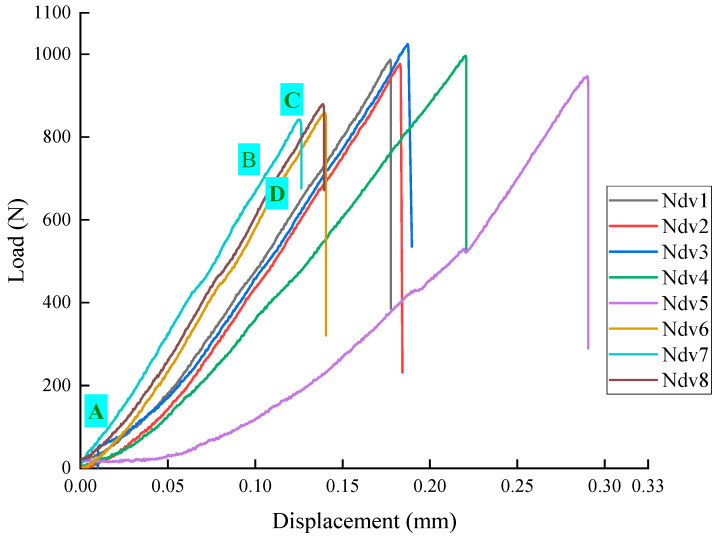
Load–displacement curves of shale Ndv 1–8 specimens.

Figure 5 presents the load–displacement curves for the Ndv1–8specimens. As the displacement increases during testing, the loads for all NSCB specimens initially rise and reach a peak value, then drop sharply. After reaching the peak load, the shale specimens fail abruptly with a distinct cracking sound, indicating brittle failure under the three-point bending load. The load–displacement curve for the semi-disk shale can be divided into four main stages: the compaction stage (segment OA in Figure 5), the elastic stage (segment AB in Figure 5), the strengthening stage (segment BC in Figure 5), and the failure stage (segment CD in Figure 5). Variability is observed across different shale specimens, indicating that the heterogeneity of the shale strongly affects its mechanical behavior.

Table 2 shows the peak load and peak displacement data for the Nah specimens in the three-point bending test. The load ranges from 859.16 N to 1024.66 N, with an average value of 938.84 N. The displacement ranges from 0.13 mm to 0.29 mm, with an average value of 0.28 mm.

**Figure 6:**
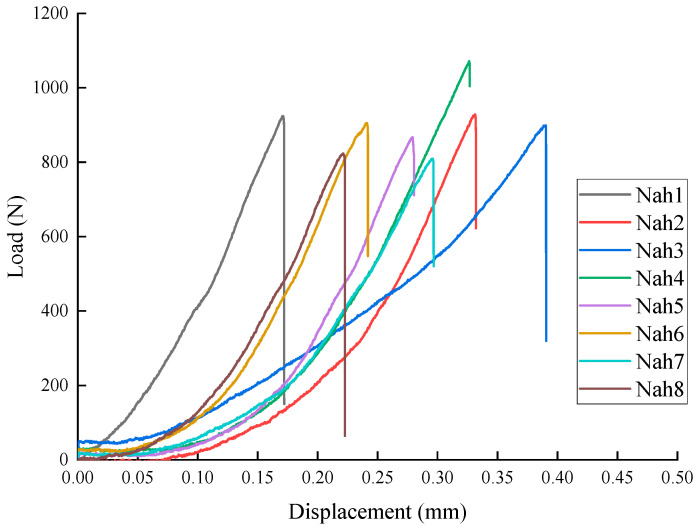
Load–displacement curves of shale Nah1–8 specimens.

Figure 6 presents the load–displacement curves for the Nah 1–8 specimens. Table 3 shows the peak load and peak displacement data for the Nah specimens in the three-point bending test. The load ranges from 809.24 N to 1071.74 N, with an average value of 903.50 N. The displacement ranges from 0.17 mm to 0.39 mm, with an average value of 0.28 mm. It can be seen that, compared to the Ndv case shown in Figure 5 and Table 2, the peak loads of the Nah specimens are lower, and the nonlinearity is more pronounced.

**Figure 7:**
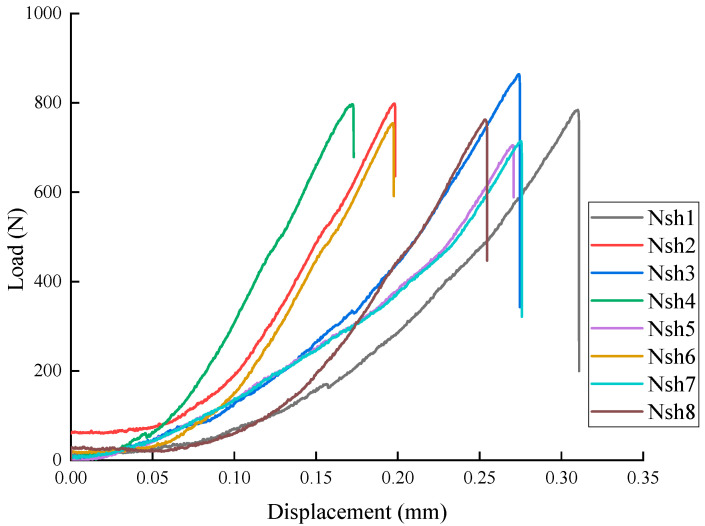
Load–displacement curves of shale Nsh1–8 specimens.

Figure 7 presents the load–displacement curves for the Nsh 1–8 specimens. Table 4 displays the peak load and peak displacement data for the Nsh specimens during the three-point bending test. The peak load values range from 705.26 N to 864.32 N, with an average of 772.61 N. The peak displacement values range from 0.17 mm to 0.31 mm, with an average of 0.24 mm. It can be seen that the peak load of the Nsh specimens is lower than that of the Nah specimens.

Through a synthesized analysis of Figure 5, Figure 6 and Figure 7 ‌in conjunction with‌ Table 2, Table 3 and Table 4, it is demonstrated that Ndv (Figure 5, Table 2) and Nah (Figure 6, Table 3) specimens exhibit higher peak loads, while Nsh (Figure 7, Table 4) specimens display lower peak loads. This difference arises from the impact of bedding direction on crack propagation. The bedding of the Nsh specimens is parallel to the loading direction (0°), causing the bedding along the crack propagation path to be more prone to cracking, resulting in the lowest peak load for Nsh specimens. In contrast, the bedding orientations of the Ndv and Nah specimens are perpendicular to the loading direction, meaning that crack propagation faces more resistance due to the misalignment between the preset crack and the loading direction, leading to higher peak loads compared to Nsh specimens.

### 2.4. Tensile Strength and COD

**Figure 8:**
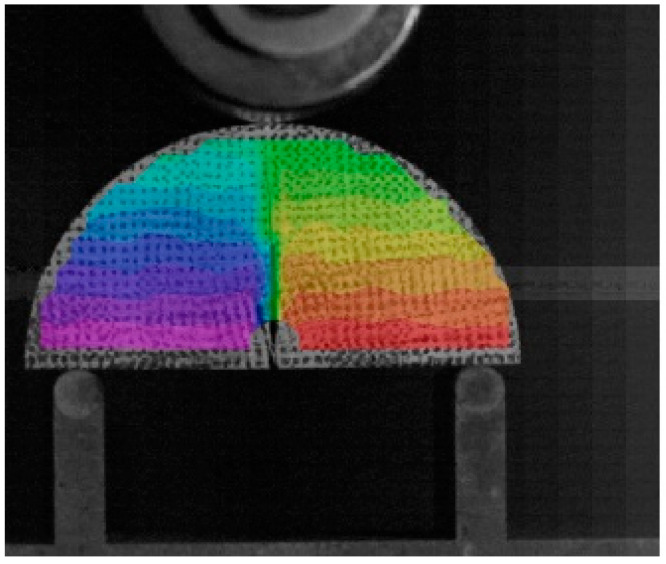
Displacement contour map of nsh8.

The scattered points on the deformed NSCB specimens were spatially reconstructed using DIC techniques. The obtained displacement field data were then smoothed. Figure 8 shows the displacement contour map of the Nsh8 specimen obtained through image processing techniques.

The changes in the positions of the scattered points at the notch before and after loading were exported, and the COD of the specimen notch was calculated using the equation:

(1) $$U=l_{i}-l_{1}$$

in which $l_{1}$ and $l_{i}$, respectively, represent the positions of the scattered points, and the subscript i is the ordinal number of the photos taken by the camera. Taking the first photo when i=1 as the reference state, $l_{1}$ represents the original positions of the features in the first photo, and the positions of the same features in the *i*-th photo are denoted as $l_{i}$.

The tensile strength of the specimens can be obtained through the formula recommended by the ISRM [35]:

(2) $$\sigma_{\max}=\frac{3S\cdot P_{\max}}{2H\cdot {\left(R-a\right)}^{2}}$$

in which $\sigma_{\max}$ is the tensile strength, S is the distance between the two support points at the bottom of NSCB (S is 40 mm which has been mentioned in Section 2.2), $P_{\max}$ is the peak load, H is the thickness of the specimen, R is the radius of the specimen, and a is the notch depth.

Referring to [36], the tensile stiffness can be obtained through the following formula:

(3) $$N=\frac{\sigma_{\max}}{\delta_{IC}}$$

where N is the tensile stiffness, and $\delta_{IC}$ is the notch opening displacement calculated according to Equation (1) when the load reaches the peak load.

From Table 5, it is evident that the tensile strengths of the Ndv specimens with vertical bedding range from 14.49 MPa to 20.12 MPa, and the tensile stiffness ranges from 30.19 MPa/mm to 159.45 MPa/mm. For Nah specimens with parallel bedding, the tensile strengths range from 16.54 MPa to 21.41 MPa, and the tensile stiffness ranges from 21.48 MPa/mm to 194.63 MPa/mm. The tensile strengths of the Nsh specimens range from 13.26 MPa to 19.78 MPa, and the tensile stiffness ranges from 26.47 MPa/mm to 126.38 MPa/mm. The tensile strengths of the Nsh and Nah specimens with parallel bedding are both lower than those of the Ndv specimens with vertical bedding.

These data reveal the significant impact of bedding orientation on the mechanical properties of shale, with specimens Nah and Nsh, which have parallel bedding, exhibiting lower tensile strengths compared to specimens Ndv with vertical bedding. Such differences in performance are a direct manifestation of the anisotropy of shale, where the mechanical response of the material varies in different directions, primarily due to the inhomogeneity in internal mechanical properties caused by the bedding structure. Understanding this anisotropy is of great practical significance for predicting the behavior of shale in actual engineering applications, such as the propagation pattern of fractures during hydraulic fracturing processes. Therefore, the in-depth study of the anisotropic characteristics of shale is crucial for optimizing engineering design and improving operational efficiency.

## 3. Evaluation of Shale Anisotropy Parameters

The calculation of shale fracturing or crack propagation requires parameters such as elastic modulus, fracture toughness, and energy release rate. Based on the results of the three-point bending tests in Section 2, the methods for acquiring each parameter are presented below.

### 3.1. Elastic Modulus

**Figure 9:**
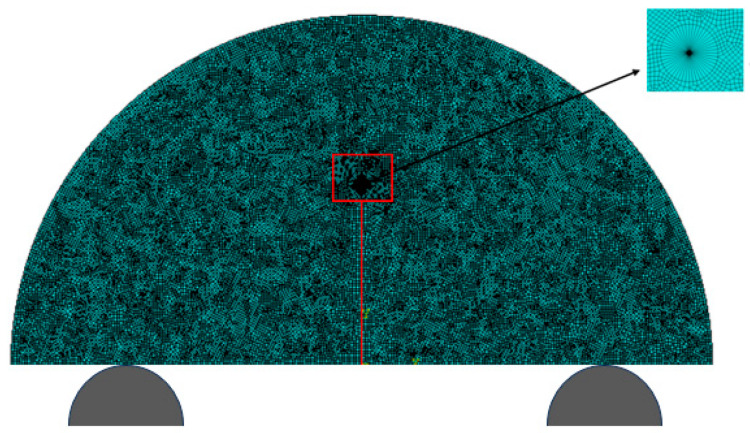
An NSCB finite element model.

To determine the elastic modulus of the specimens, a finite element model was established, as shown in Figure 9. In this paper, the finite element software ABAQUS (https://www.3ds.com/products/simulia/abaqus, accessed on 27 March 2025) was used. The size of the numerical model corresponds to the actual size of the NSCB specimens, with a radius of R=25 mm and a notch depth of a=13 mm. The model consists of 13,432 quadrilateral elements. The supports are treated as two rigid bodies, while the specimen is modeled as a linear-elastic body, and the specimen and supports are set as rigid contact. Referring to [37], the Poisson’s ratio ν is taken as 0.25. Given the initial anisotropic elastic modulus, the shear modulus in the *i*-*j* plane is calculated as follows:

(4) $$\frac{1}{G_{ij}}=\frac{1}{E_{i}}+\frac{1+2v}{E_{j}}\;\;i,j=1,2,3$$

Due to the presence of rough microstructures and pre-existing microscopic defects in shale, as well as multiple stable micro-fracture events prior to peak load, accurately simulating the closure of initial cracks and pores in the numerical model is challenging. During the initial loading stage, the test specimens exhibit concave and nonlinear deformation, which cannot be simulated directly in the model. Therefore, the principle is to adjust the simulation so that the elastic deformation stage matches the slope of the experimental curve, thereby determining the anisotropic elastic modulus.

**Figure 10:**
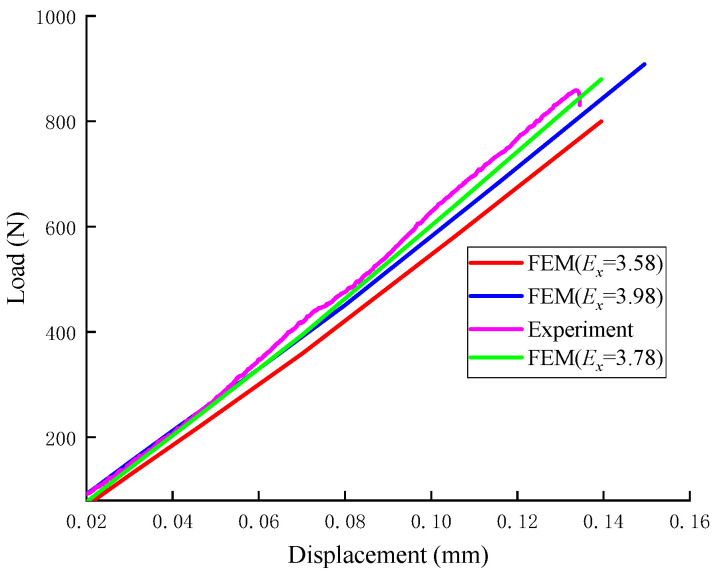
Load–displacement curves for the Ndv8 specimen with different elastic modulus.

Figure 10 shows the load–displacement curve with different elastic moduli. The elastic modulus was adjusted to 3.58 (represented by the red curve), 3.98 (blue curve), and 3.78 (green curve), with the Ndv-8 experimental curve shown for reference. The results demonstrated noticeable differences in the load–displacement curves corresponding to the different elastic modulus values, highlighting the significant influence of the elastic modulus on the mechanical response of the material.

**Figure 11:**
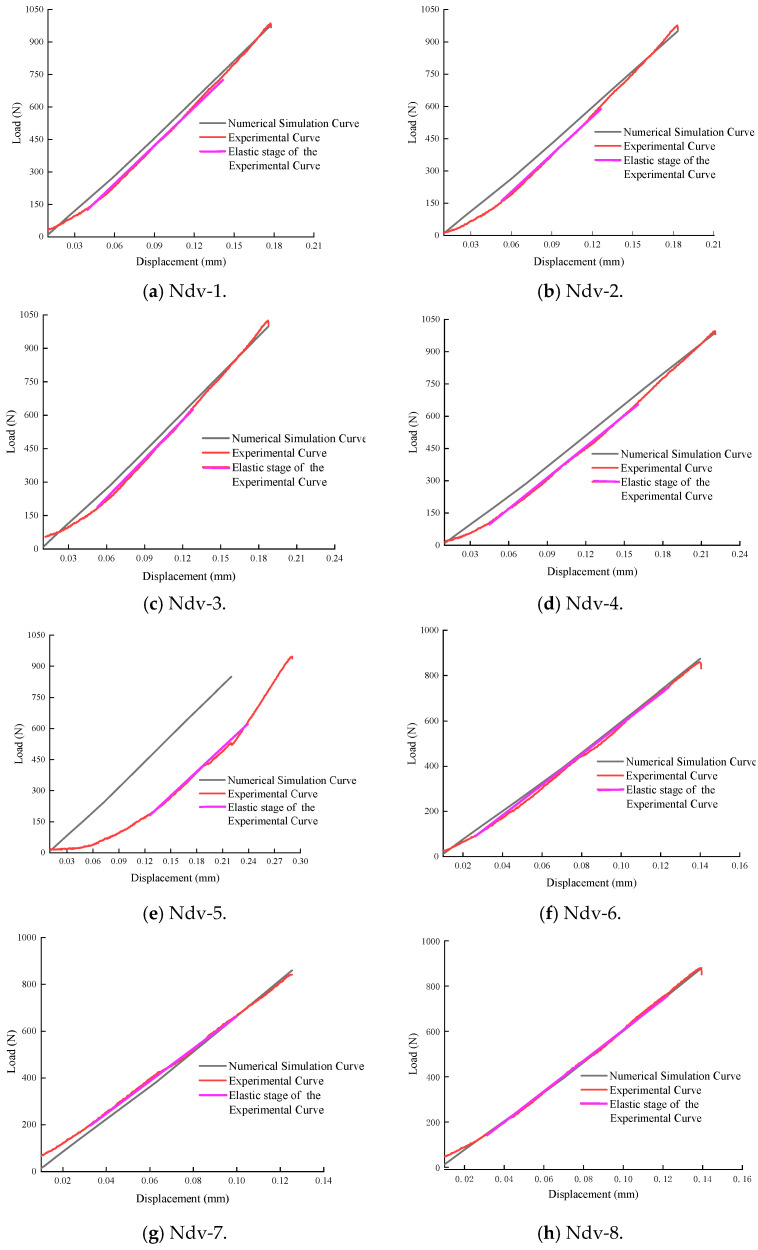
The load–displacement curves by finite element method and Ndv experiments.

**Figure 12:**
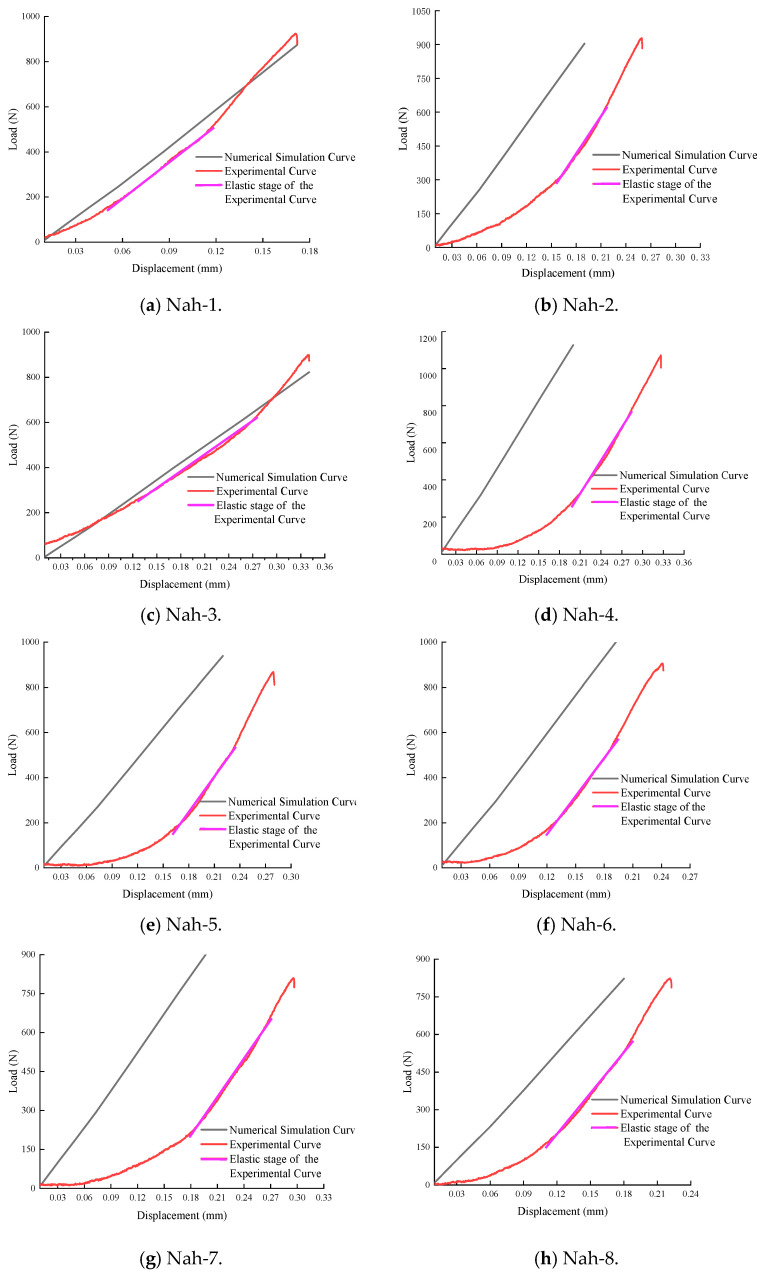
The load–displacement curves by finite element method and Nah experiments.

**Figure 13:**
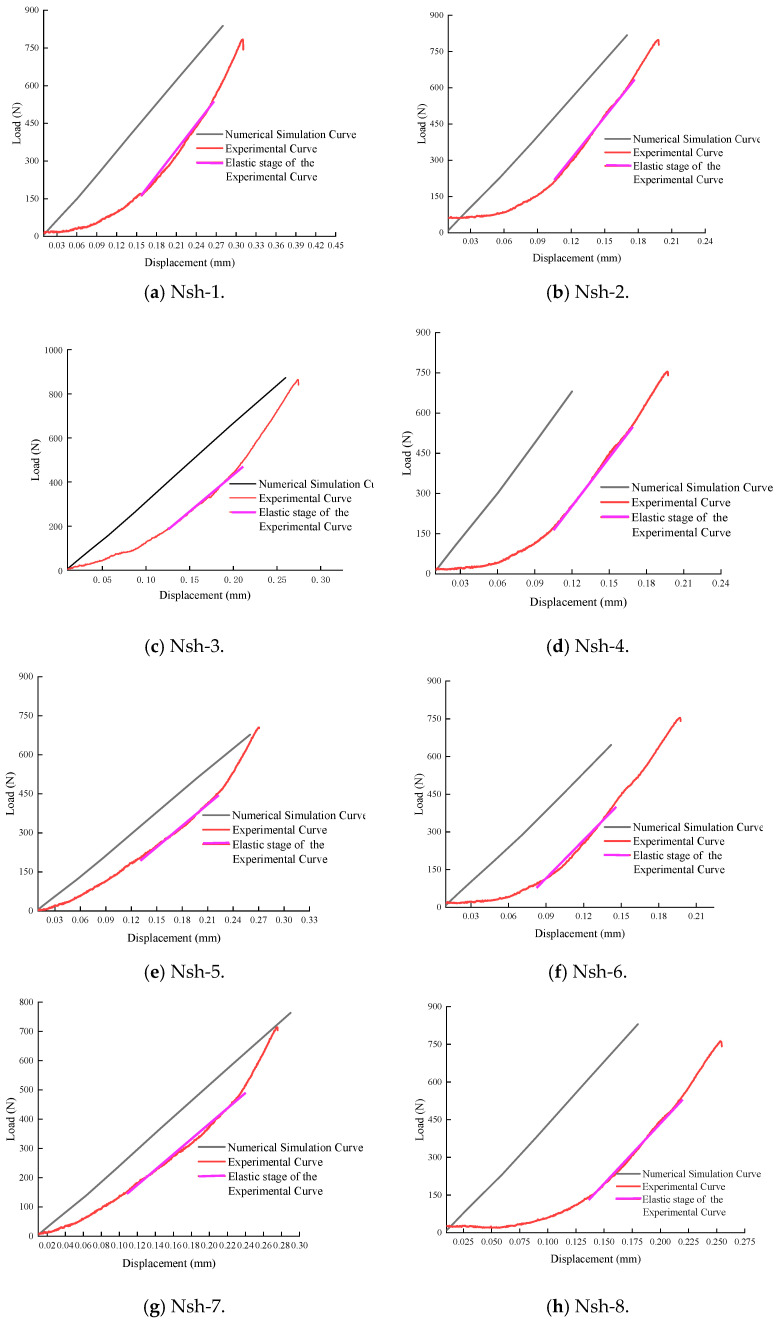
The load–displacement curves by finite element method and Nsh experiments.

In the inversion analysis of elastic modulus, within the elastic region of the load–displacement curve, if the experimental curve and the finite element model curve have the same slope, it indicates that the elastic behaviors are similar, meaning their elastic moduli are equivalent. The value of the elastic modulus is adjusted until the slope of the simulated curve closely matches that of the experimental curve. This process yields an elastic modulus that aligns with the experimental data. The adjusted load–displacement curves from the finite element method and the experiments for the Ndv, Nah, and Nsh specimens are shown in Figure 11, Figure 12 and Figure 13. The numerical simulation curve (black line), experiment curve (red line), and the pink line of the elastic stage of the experiment cure can all be observed. It can be seen that the simulation curves are almost parallel to the pink line of the elastic stage of the experiment cure. The corresponding elastic modulus for each specimen can be obtained.

The elastic modulus and shear modulus of specimens with different bedding orientations obtained from the simulations are presented in Table 6, Table 7 and Table 8.

In Table 6, the elastic modulus values *E_x_* for the Ndv specimens are determined to range from 3.09 GPa to 3.99 GPa, averaging at 3.85 GPa, and *E_y_* from 3.00 GPa to 3.93 GPa, averaging at 3.60 GPa. For the Nah specimens, shown in Table 7, *E_y_* ranges from 3.01 GPa to 3.95 GPa, with an average of 3.44 GPa, and *E_z_* from 3.04 GPa to 3.72 GPa, averaging at 3.32 GPa. In Table 8, *E_z_* for the Nsh specimens exhibit a range from 3.10 GPa to 3.97 GPa, averaging at 3.31 GPa, and *E_x_* from 3.11 GPa to 3.99 GPa, with an average of 3.64 GPa. The differences in average elastic modulus values across the different series specimens can be observed, with the *E_x_* showing a 0.21 GPa difference between the Ndv and Nsh specimen, the *E_y_* direction displaying a 0.16 GPa difference between the Ndv and Nah specimen, and the *E_z_* direction indicating a 0.01 GPa difference between the Nsh and Nah specimen. Such differences may be due to the internal structure of the material, the arrangement of bedding planes, or other microstructural characteristics, and they represent the anisotropic properties of the material.

Averaging values of $E_{x}$, $E_{y}$, and $E_{z}$ in Table 6, the average value of $E_{x}$ is 3.75 GPa, $E_{y}$ is 3.52 GPa, and $E_{z}$ is 3.31 GPa, respectively. Regarding i,j=1,2,3 in Equation (4) as x,y,z, based on Equation (4) and the existing parameters of the elastic modulus and Poisson’s ratio, the calculated value of $G_{xy}$ is 1.45 GPa, the value of $G_{xz}$ is 1.39 GPa, and the value of $G_{yz}$ is 1.36 GPa. The elastic modulus obtained using the inversion method is consistent with the measured results from the Brazilian splitting tests in reference [38]. This confirms that the inversion technique used in this study can effectively predict the elastic properties of shale.

The differences in the elastic modulus in various directions serve as a clear indication of the material’s anisotropy. This anisotropic characteristic is crucial for predicting the behavior of materials under different loading conditions, such as in hydraulic fracturing or other geological engineering applications.

### 3.2. Fracture Toughness

For the NSCB specimens, the ISRM has provided the calculation formula for fracture toughness [18].

(5) $$K_{IC}=\frac{P_{max}\sqrt{\pi a}}{2RH}Y^{\prime }$$

in which, $K_{\mathrm{IC}}$ is the fracture toughness, $\;Y^{\prime }$ is the dimensionless stress intensity factor. Zhao et al. [31] and Zheng et al. [39] used the calculation formula of the dimensionless stress intensity factor $Y^{\prime }$ of rock recommended by the ISRM to calculate $\;Y^{\prime }$ of shale, that is as follows:

(6) $$Y^{\prime }=-1.297+9.516\left(\frac{S}{2R}\right)-\left(0.47+16.457\left(\frac{S}{2R}\right)\right)\left(\frac{a}{R}\right)+\left(1.071+34.401\left(\frac{S}{2R}\right)\right){\left(\frac{a}{R}\right)}^{2}$$

Finite element analysis of NSCB specimens is also always employed to calculate the values of stress intensity factors [40,41]. Stress intensity factor $\;Y^{\prime }$, using the finite element method, can be evaluated as

(7) $$Y^{\prime }\left(\frac{a}{D},\frac{S}{D},\alpha ,E_{x},E_{y},v,G_{x},G_{y}\right)=\frac{K_{\mathrm{tip}\;}DH}{P_{\max}\sqrt{\pi a}}$$

In Equation (7), $K_{\mathrm{tip}\;}$ is the stress intensity factor at the crack tip when the vertical load reaches the peak load.

By substituting the NSCB experimental data into Equations (5) and (6), the fracture toughness and stress intensity factor of the NSCB specimens are calculated and presented in Table 9. A three-point bending finite element model, shown in Figure 9, was used to match the actual size and loading conditions of the NSCB specimens. Equation (7) is used to calculate stress intensity factor $K_{\mathrm{tip}\;}$ of the specimens. Then, $K_{\mathrm{tip}\;}$ is substituted into Equation (7) to calculate $\;Y^{\prime }$, finally obtaining the result $K_{\mathrm{1C}}$, which is listed in Table 9. It is evident from Table 9 that the fracture toughness obtained from the finite element model is slightly lower than that obtained from the ISRM-recommended formula. Combining the results from both methods, as calculated by Equation (6) and the finite element method, it is evident that the Ndv specimens exhibit higher fracture toughness, followed by the Nah specimens. In contrast, the Nsh specimens, due to their greater propensity for propagation, have the lowest fracture toughness.

In Ref. [41], the fracture toughness values $K_{1C}$ for shale were tested within a similar range of 32 to 42 $\left(\mathrm{MPa}\cdot \sqrt{\mathrm{mm}}\right)$. Our study further expanded on their findings by incorporating additional tests with specimens featuring different bedding orientations.

### 3.3. Energy Release Rate

The energy release rate is a critical parameter for determining whether a crack will propagate and plays a crucial role in the numerical simulations of crack propagation. Several methods exist for calculating the energy release rate of NSCB specimens [27]. Among these, the energy release rate can be derived from the fracture toughness as follows [42,43,44]:

(8) $$G_{1C}=\frac{K_{IC}^{2}}{E^{*}}$$

(9) $$E^{*}=\sqrt{\frac{2E_{i}E_{j}\lambda^{1/2}}{1+\rho }}$$

where

(10) $$\lambda =\frac{E_{i}}{E_{j}}$$

(11) $$\rho =\frac{\sqrt{E_{i}E_{j}}}{2G_{ij}}-\sqrt{\nu_{ij}\nu_{ji}}$$

The fracture toughness $K_{1C}$ can be obtained from Section 3.2, and $v_{ij}=v_{ji}=0.25$. From Section 3.1, the average values of $E_{x}$, $E_{y}$, and $E_{z}$ are 3.75 GPa, 3.52 GPa, and 3.31 GPa, respectively.

The values are substituted to calculate the energy release rate and shown in Table 10, Table 11 and Table 12 as follows. Table 10 provides the peak energy release rate data for the Ndv specimens during the fracture test. The energy release rate $G_{IC}$ values range from 0.42 N/mm to 0.82 N/mm, with an average value of 0.55 N/mm.

Table 11 provides the peak energy release rate data for the Nah specimens in the three-point bending test. The energy release rate $G_{IC}$ values range from 0.38 N/mm to 0.79 N/mm, with an average of 0.52 N/mm. Compared to the Ndv specimens, the Nah specimens have a slightly lower average energy release rate.

Table 12 presents the peak energy release rate data for the Nsh specimens during the three-point bending test. The energy release rate $G_{IC}$ values vary from 0.31 N/mm to 0.46 N/mm, with an average value of 0.31 N/mm. It can be observed that the peak energy release rate of the Nsh specimens is lower than that of the Nah specimens.

The energy release rates in Table 10, Table 11 and Table 12 exhibit obvious anisotropy of shale. This indicates that the Ndv and Nah specimens require more energy during the fracture process, demonstrating higher fracture toughness, while the Nsh specimens, being more prone to propagation, have relatively lower fracture energy and weaker resistance to fracture.

## 4. Numerical Simulation of Three-Point Bending Fracture Behavior of Shale Anisotropy

**Figure 14:**
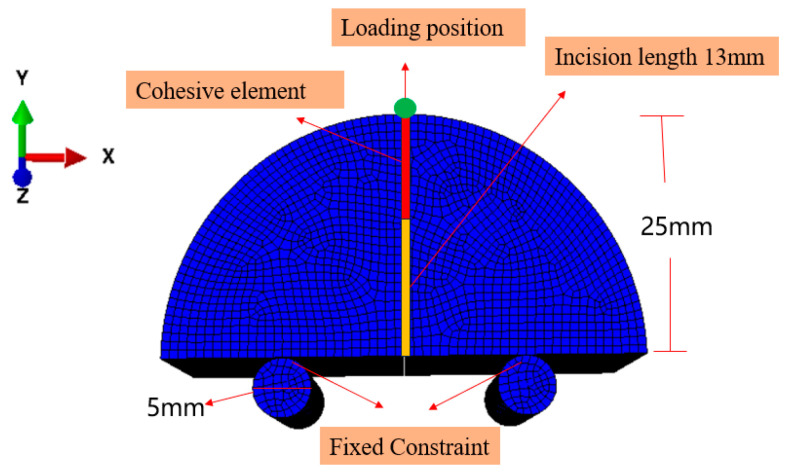
Shale three-point bending model.

The parameters detailed in the preceding sections serve as essential inputs for the simulation of crack propagation. Based on the FEM model for Ndv8 specimens generated using ABAQUS in Section 3.1, cohesive elements were incorporated into the finite element model described in Section 3, creating a new NSCB finite element model (shown in Figure 14). The Ndv8 specimen was selected for the simulation. According to the anisotropic parameters of shale obtained in Section 2 and Section 3, the specific simulation parameters are shown in Table 13.

**Figure 15:**
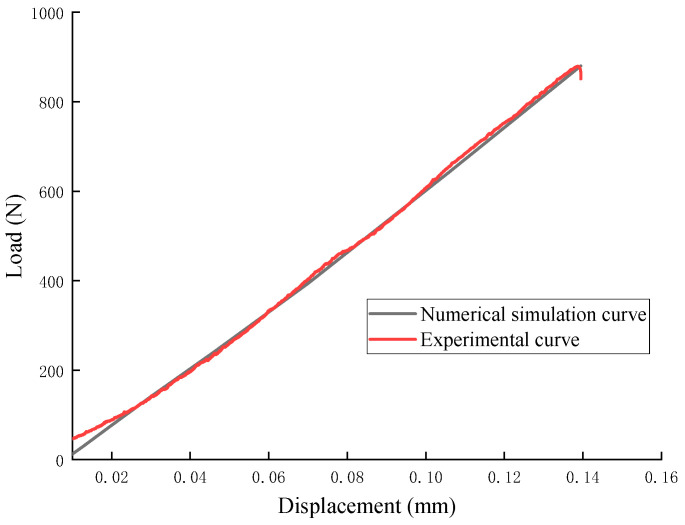
Load–displacement curves from the numerical model and experiments.

**Figure 16:**
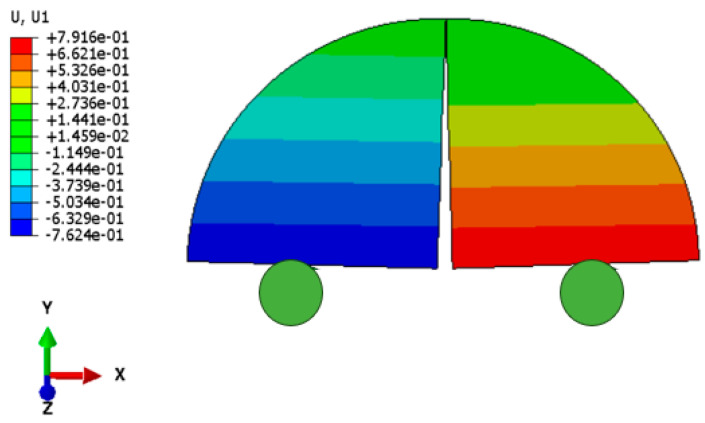
Contour nephogram of lateral displacement (mm).

Figure 15 presents a comparison between the simulation and experimental load–displacement curves. It can be observed that the finite element model’s load–displacement curves align well with the experimental data. Figure 16 also shows the displacement contour cloud when the load reaches its peak, indicating that the adopted finite element model and parameters are reasonable and reliable.

## 5. Conclusions

Through shale NSCB three-point bending tests, anisotropic parameters such as the fracture toughness, fracture energy, and energy release rate for shale in the Changning area of Yibin, Sichuan, were obtained to investigate crack initiation and propagation in shale. The conclusions are as follows:

(1) The load–displacement curves, CODs, tensile strength, and tensile stiffness of Ndv, Nsh, and Nah NSCB specimens were obtained. The results indicate that crack propagation in Ndv and Nah specimens is more difficult than in Nsh specimens, where the notch is parallel to the bedding.

(2) By fitting the load–displacement curves, the anisotropic elastic modulus and fracture toughness parameters of NSCB specimens were evaluated. The evaluated elastic modulus obtained via the inversion method is consistent with the measured results from the literature. The stress intensity factors from the formula recommended by the literature are slightly higher than those derived from the finite element method.

(3) The energy release rate was calculated using fracture toughness. The study showed that both the fracture toughness and the energy release rate of shale are greater in the direction perpendicular to the bedding than in the parallel bedding direction.

(4) Parameters such as elastic modulus, opening displacement, and energy release rate for the Ndv-8 specimen were input into the NSCB numerical simulation. The load–displacement curve obtained from the simulation showed good agreement with the experimental results.

(5) The systematic and comprehensive method proposed in this study for determining anisotropic fracture parameters in shale provides a robust framework. The derived fracture parameters can serve as critical inputs to validate predictive simulations of hydraulic fracturing in shale reservoirs. The results also can assist engineers in devising more rational fracturing plans, reducing the risks of wellbore instability and thereby enhancing both the safety and economic efficiency of shale gas extraction.

## Figures and Tables

**Table 1:** Physical parameters of shale NSCB specimens.

Mode	Specimens	Thickness (mm)	Radius (mm)	Notch Depth (mm)	Mass (g)	Volume (cm^3^)	Density (g/cm^3^)
Divider	Ndv-1	20.3	24.7	12.2	49.06	19.44	2.52
	Ndv-2	20.3	25.0	12.2	50.88	19.92	2.55
	Ndv-3	20.1	24.9	12.1	49.77	19.56	2.54
	Ndv-4	20.1	25.0	12.6	50.16	19.72	2.54
	Ndv-5	20.0	24.4	12.6	48.76	18.69	2.60
	Ndv-6	20.2	24.6	12.5	48.18	19.19	2.51
	Ndv-7	20.4	24.2	12.2	48.90	18.75	2.60
	Average	20.2	24.6	12.3	49.31	19.25	2.56
Arrester	Nah-1	20.1	24.9	12.6	49.29	19.56	2.51
	Nah-2	20.3	25.1	12.4	50.24	20.08	2.50
	Nah-3	20.3	25.0	12.5	50.19	19.92	2.52
	Nah-4	20.2	24.8	12.9	50.56	19.51	2.59
	Nah-5	20.2	24.2	12.5	48.24	18.57	2.59
	Nah-6	20.2	24.5	13.0	47.57	19.03	2.50
	Nah-7	20.3	24.4	12.9	48.57	18.97	2.56
	Nah-8	20.1	24.0	12.9	47.52	18.18	2.61
	Average	20.2	24.6	12.7	49.02	19.23	2.55
Splitter	Nsh-1	20.3	24.8	12.5	49.87	19.60	2.54
	Nsh-2	20.3	25.0	12.3	50.82	19.91	2.55
	Nsh-3	20.1	25.0	12.2	49.87	19.72	2.52
	Nsh-4	20.2	25.2	12.5	48.71	20.13	2.41
	Nsh-5	20.2	24.2	13.0	47.73	18.57	2.57
	Nsh-6	20.1	24.1	12.9	48.00	18.32	2.62
	Nsh-7	20.8	24.3	12.8	49.43	19.28	2.56
	Nsh-8	20.3	24.5	12.8	47.53	19.13	2.48
	Average	20.3	24.6	12.6	48.89	19.33	2.53

**Table 2:** The peak load and displacement of shale Ndv1–8 specimens.

Specimens	Ndv-1	Ndv-2	Ndv-3	Ndv-4	Ndv-5	Ndv-6	Ndv-7	Ndv-8	Average
Peak Load (N)	986.66	976.22	1024.66	996.38	946.68	859.16	841.78	879.14	938.84
Peak displacement (mm)	0.18	0.18	0.19	0.22	0.29	0.14	0.13	0.14	0.18

**Table 3:** The peak load and displacement of shale Nah1–8 specimens.

Specimens	Nah-1	Nah-2	Nah-3	Nah-4	Nah-5	Nah-6	Nah-7	Nah-8	Average
Peak Load (N)	924.24	928.06	899.02	1071.74	867.24	905.06	809.24	823.38	903.50
Peak displacement (mm)	0.17	0.33	0.39	0.33	0.28	0.24	0.30	0.22	0.28

**Table 4:** The peak load and displacement of shale Nsh1–8 specimens.

Specimens	Nsh-1	Nsh-2	Nsh-3	Nsh-4	Nsh-5	Nsh-6	Nsh-7	Nsh-8	Average
Peak Load (N)	784.36	798.42	864.32	797.14	705.26	754.42	714.26	762.68	772.61
Peak displacement (mm)	0.31	0.20	0.27	0.17	0.27	0.20	0.28	0.25	0.24

**Table 5:** The tensile strength and tensile stiffness calculated for the NSCB specimens.

Specimens	$\mathrm{Notch}\;\mathrm{Opening}\;\mathrm{Displacement}\;(\mathrm{mm})\;\delta_{IC}$	$\mathrm{Tensile}\;\mathrm{Strength}\;\sigma_{\max}$ (Mpa)	Tensile Stiffness N (Mpa/mm)
Ndv-1	0.60	18.66	31.10
Ndv-2	0.47	17.48	37.19
Ndv-3	0.42	17.53	41.74
Ndv-4	0.48	14.49	30.19
Ndv-5	0.63	20.12	31.94
Ndv-6	0.11	17.54	159.45
Ndv-7	0.30	17.24	57.47
Ndv-8	0.46	18.48	40.17
Average	0.46	17.69	53.66
Nah-1	0.45	18.14	40.31
Nah-2	0.77	16.54	21.48
Nah-3	0.33	16.71	50.64
Nah-4	0.11	21.41	194.63
Nah-5	0.60	19.28	32.13
Nah-6	0.35	20.17	57.62
Nah-7	0.58	18.09	31.19
Nah-8	0.35	19.85	56.71
Average	0.47	18.77	60.59
Nsh-1	0.49	15.17	30.96
Nsh-2	0.34	13.26	39.00
Nsh-3	0.13	16.43	126.38
Nsh-4	0.37	14.68	39.68
Nsh-5	0.29	13.93	48.03
Nsh-6	0.31	19.78	63.81
Nsh-7	0.58	15.35	26.47
Nsh-8	0.26	16.26	53.48
Average	0.35	15.61	44.60

**Table 6:** The elastic modulus of Ndv specimens by inversion.

Elastic Modulus	Ndv-1	Ndv-2	Ndv-3	Ndv-4	Ndv-5	Ndv-6	Ndv-7	Ndv-8	Average
$E_{x}$ (Gpa)	3.99	3.77	3.95	3.09	3.39	3.86	3.89	3.78	3.85
$E_{y}$ (Gpa)	3.63	3.43	3.09	3.00	3.06	3.90	3.93	3.86	3.60

**Table 7:** The elastic modulus of Nah specimens by inversion.

Elastic Modulus	Nah-1	Nah-2	Nah-3	Nah-4	Nah-5	Nah-6	Nah-7	Nah-8	Average
$E_{y}$ (Gpa)	3.63	3.33	3.01	3.95	3.31	3.65	3.46	3.21	3.44
$E_{z}$ (Gpa)	3.48	3.19	3.04	3.72	3.45	3.42	3.21	3.09	3.32

**Table 8:** The elastic modulus of Nsh specimens by inversion.

Elastic Modulus	Nsh-1	Nsh-2	Nsh-3	Nsh-4	Nsh-5	Nsh-6	Nsh-7	Nsh-8	Average
$E_{z}$ (Gpa)	3.10	3.27	3.13	3.97	3.18	3.11	3.22	3.39	3.31
$E_{x}$ (Gpa)	3.96	3.64	3.16	3.99	3.11	3.64	3.84	3.78	3.64

**Table 9:** Stress intensity factor and fracture toughness of NSCB specimens.

**Specimens**	Equation (7)		Finite Element Model	
	$Y^{\prime }$	$K_{\mathrm{IC}}\;(\mathrm{MPa}\cdot \sqrt{\mathrm{mm}})$	$Y^{\prime }$	$K_{\mathrm{1C}}\;(\mathrm{MPa}\cdot \sqrt{\mathrm{mm}})$
Ndv-1	7.16	44.92	6.86	41.77
Ndv-2	6.96	42.61	6.81	40.57
Ndv-3	7.02	43.58	8.41	50.45
Ndv-4	6.96	43.85	6.75	42.13
Ndv-5	7.37	45.54	6.64	40.40
Ndv-6	7.23	39.81	7.15	38.88
Ndv-7	7.52	41.10	6.97	36.82
Ndv-8	7.52	42.61	6.73	37.63
Average	7.22	43.00	7.04	41.35
Nah-1	7.02	41.44	6.43	37.35
Nah-2	6.89	40.21	6.79	38.62
Nah-3	6.96	39.53	6.28	34.86
Nah-4	7.10	48.43	7.07	48.11
Nah-5	7.52	42.61	6.47	36.21
Nah-6	7.30	42.61	7.62	44.50
Nah-7	7.37	38.54	7.45	38.73
Nah-8	7.68	41.71	6.47	35.14
Average	7.23	41.83	6.82	38.93
Nsh-1	7.09	35.46	7.51	36.66
Nsh-2	6.96	40.48	7.73	34.95
Nsh-3	6.96	38.28	7.90	42.56
Nsh-4	6.83	30.35	6.63	32.54
Nsh-5	7.52	31.62	7.00	31.01
Nsh-6	7.60	37.94	5.15	27.66
Nsh-7	7.44	33.51	7.76	34.78
Nsh-8	7.30	35.74	7.30	35.48
Average	7.21	35.55	7.12	34.07

**Table 10:** Energy release rate of Ndv specimens.

Energy Release Rate	Ndv-1	Ndv-2	Ndv-3	Ndv-4	Ndv-5	Ndv-6	Ndv-7	Ndv-8	Average
$G_{IC}$ (N/mm)	0.53	0.52	0.82	0.56	0.51	0.47	0.42	0.44	0.55

**Table 11:** Energy release rate of Nah specimens.

Energy Release Rate	Nah-1	Nah-2	Nah-3	Nah-4	Nah-5	Nah-6	Nah-7	Nah-8	Average
$G_{IC}$ (N/mm)	0.46	0.49	0.39	0.79	0.41	0.62	0.48	0.38	0.52

**Table 12:** Energy release rate of Nsh specimens.

Energy Release Rate	Nsh-1	Nsh-2	Nsh-3	Nsh-4	Nsh-5	Nsh-6	Nsh-7	Nsh-8	Average
$G_{IC}$ (N/mm)	0.39	0.36	0.46	0.33	0.31	0.27	0.36	0.38	0.31

**Table 13:** Simulation parameters for the NSCB model of Ndv8 specimen.

**Material**	**Elastic Modulus (GPa)**		Shear Modulus		Poisson’s Ratio		Energy Release Rate (N/mm)	
Shale	$E_{x}$	$E_{y}$	$E_{z}$	$G_{xy}$	$G_{xz}$	$G_{yz}$	0.25	/
	3.75	3.52	3.31	1.45	1.39	1.36		
Cohesive	3.5			/			/	0.44

## Data Availability

The original contributions presented in this study are included in the article. Further inquiries can be directed to the corresponding author.
